# Bifurcation study of a tumor-immune system with chemotherapy

**DOI:** 10.1371/journal.pone.0327304

**Published:** 2025-07-03

**Authors:** Abdelhamid Ajbar, Rubayyi T. Alqahtani, Khalid Alhumaizi

**Affiliations:** 1 Department of Chemical Engineering Department, College of Engineering, King Saud University, Riyadh, Saudi Arabia; 2 Department of Mathematics and Statistics, College of Science, Imam Mohammad Ibn Saud Islamic University (IMSIU), Riyadh, Saudi Arabia; University of the Philippines Diliman, PHILIPPINES

## Abstract

Understanding the dynamics of cancer cell growth, the interplay between tumor and immune cells, and the efficacy of chemotherapy are pivotal areas of focus in cancer research. In this regard, mathematical modeling can provide significant insights. This study re-examines a classical two-dimensional model of tumor-immune cell interactions where the tumor’s growth rate is assumed to adhere to von Bertalanffy’s model instead of the logistic model. We investigate the model both without chemotherapy and with treatment. The equilibrium points are identified, classified, and their stability analyzed. Our results reveal that the model can demonstrate a broad spectrum of behaviors, including bi-stability and multi-stability as well as regions of stable periodic behavior. We establish analytical conditions for the existence of Hopf points. Furthermore, we assess the impact of model parameters on the various behavior predicted by the model. This mathematical investigation can provide general guidance on treatment strategies.

## 1 Introduction

Cancer is still a major contributor to global mortality rates. The costs related to its treatment and management have a considerable financial impact especially on health care systems in less developed countries. According to global estimates from 2022, approximately 20 million new cases of cancer were diagnosed, resulting in close to 10 million deaths [1].

Extensive research efforts are in progress to discover new therapeutic options and to improve the performance of existing ones [2, 3]. A key element of this research is the integration of mathematical modeling in cancer research studies. Such modeling is important for multiple reasons including clarifying the growth behaviors of tumor cells [4], customizing and refining treatment protocols [5, 6], investigating drug resistance [7], and predicting the efficacy of new therapeutic interventions [8].

The numerous mathematical models proposed in the literature are characterized by differences in the number of cell types considered such as NK, $\text{CD}8^{+}\text{T}$, and $\text{CD}4^{+}\text{T}$ cells as well as the modeling techniques used which encompass ordinary differential equations, partial differential equations, fractional order models and stochastic models [9–23]. The dynamics between immune cells and cancer are commonly analogized to the interactions between predators and their preys. When activated, immune cells behave like predators, chasing after tumor cells, creating physical connections, and destroying the cells they target. An extensive examination of the similarities between tumor-immune interactions and predator-prey relationships was explored in [24].

The early research by Kuznetsov *et al*. [9] established one of the basic mathematical frameworks for understanding the interactions between tumor cells and immune cells represented through a predator-prey model. That framework comprised two ordinary differential equations and focused on two distinct cell types: effector cells which function as the predator, and tumor cells which serve as the prey. The analysis [9] indicated the existence of parameter spaces where only one type of cell could persist, regions of bi-stability and areas where dormant tumor cells could re-activate. de Pillis *et al*. [11, 12] later developed and analyzed a model of tumor-immune cells interactions. The authors showed the presence of bi-stability regions between the disease-free state and a high tumor cells concentration driven by a number of mechanisms such as saddle-node and transcritical bifurcations. Alternatively, López *et al*. [14] proposed and studied a different tumor-immune cells model with the presence of chemotherapy, and revealed similar results to the work in [11, 12]. On the other hand, Makhlouf *et al*. [16] simulated an ODE model that included the interactions among tumor cells, circulating lymphocytes, $\text{CD}8^{+}$ T cells, $\text{CD}4^{+}$ T cells, and natural killer cells, taking into account the effects of chemotherapy. Song *et al*. [21] explored the stability of a model that depicted linear interactions between tumor cells and immune cells, drawing attention to the significant contributions of natural killer cells and cytotoxic T lymphocytes in the immune surveillance system. Recently, Bashkirtseva *et al*. [20] updated the original system [9] by integrating chemotherapy treatment. The authors [20] analyzed the influence of chemotherapy dose and revealed the existence of areas of periodic behavior as well as static states. A number of recent studies (e.g. [18]) have also investigated the stability of tumor-immune models considering the effects of monoclonal antibody-targeted chemotherapy. This treatment approach may lead to fewer side effects in comparison to conventional chemotherapy methods.

An essential element of mathematical modeling regarding the interactions between tumor cells and immune cells is the selection of the most appropriate growth rate for tumor cells. Several growth models were proposed and analyzed in the literature [4]. These models include linear, logistic, Mendelsohn, exponential, Gompertz, Surface, and Bertalanffy models [4]. It is generally acknowledged that the choice of growth model is dependent on the specific type of tumors. In the context of general mathematical analysis, the literature (e.g. [9, 11, 12, 14–16, 20, 21]) has largely relied on the logistic growth rate. This reliance is partly due to the ease of mathematical analysis afforded by the logistic function, although it is not necessarily the most accurate representation of tumor growth [4].

The first objective (and novelty) of the the current work is to extend the early work of Kuznetsov *et al*. [9] to the von Bertalanffy growth rate [25]. The von Bertalanffy model suggests that the growth is proportional to surface area and also incorporates a decline in tumor size due to cell death. It was established that this growth model offers one of the best fits for understanding tumor growth in human subjects [4, 26–28].

The second objective of this study is to include the effects of chemotherapy in the proposed model and to examine the effect of interactions between the model biological (intrinsic) parameters and those associated with chemtoherapy on the dynamics of the model. We determine the equilibria of the model and investigate analytically and numerically bifurcation phenomena in the model. It turns out that complex and rich dynamics are uncovered, and practical diagrams are constructed to delineate the effect of the different parameters on the outcome of the treatment. As far as we know, there has been no prior research that studied specifically the dynamics of competition based on the von Bertalanffy growth rate.

The remaining sections of the paper are structured as follows. Sect 2 introduces the model while Sect 3 discusses the selection of model parameters. Sect 4 encompasses the derivation and classification of equilibria. An examination of the model in the absence of chemotherapy is conducted in Sect 5. Sect 6 focuses on the investigation of the model incorporating chemotherapy treatment, culminating in a discussion in the final section.

## 2 The mathematical model

The model put forth for tumor-immune interactions is based on the formulation described in references [9, 20]. It includes two classes: Class *E*, which represents the population of effector cells (predators), and Class *T* which represents the population of tumor cells (prey). The model equations are as follows:

$$\frac{dE}{dt}=s+\frac{pET}{g+T}-mET-dE-\frac{k_{E}Ev}{1+h_{1}E}$$ (1)

$$\frac{dT}{dt}=\alpha T^{2/3}-\beta T-nET-\frac{k_{T}Tv}{1+h_{2}T}.$$ (2)

The variables E (cell) and T (cell) represent the populations of effector cells and tumor cells, respectively. Effector cells increase at a constant growth rate of $s\;(\frac{cell}{day})$ and they decrease at a constant death rate of $d\;(\frac{1}{day})$. The growth of effector cells is affected by their interaction with tumor cells, following a Michaelis-Menten growth rate described by the term $\frac{pET}{g+T}$, where $p\;(\frac{1}{day})$ indicates the maximum recruitment potential of effector cells, and g (cell) denotes the steepness coefficient of the recruitment curve. Additionally, the decline in effector cells is influenced by their interactions with tumor cells at a rate of $m\;(\frac{1}{cell.day})$.

The growth of tumor cell populations (Eq 2) is modeled using the von Bertalanffy growth model [4, 25–28], where the parameters $\alpha \;(\frac{cell^{1/3}}{day})$ and $\beta \;(\frac{1}{day})$ serve as coefficients for an isolated tumor cell population. The exponent (2/3) indicates that the growth is proportional to the surface area, which correlates to volume raised to the power of (2/3). Additionally, the energy required for maintenance is directly proportional to the cell count and is represented by βT. Reduction of tumor cells by immune cells is carried out at a rate of $n\;(\frac{1}{cell.day})$.

The effect of chemotherapy on both cells is given by the non-linear terms $\frac{k_{E}Ev}{1+h_{1}E}$ and $\frac{k_{T}Tv}{1+h_{2}T}$, expressions inspired by the work in [20]. The term $v\;(\frac{mg}{BSA.day})$ represented the daily dose of drug, where *BSA*(*m*^2^) indicates the body surface area. The BSA method of dosing is commonly used in oncology to calculate drug dosages, as it provides a more accurate dosing than weight-based methods. The parameter $k_{E}\;(\frac{m^{2}}{kg})$ denotes the fractional effector cells kill by chemotherapy while $k_{T}\;(\frac{m^{2}}{kg})$ denotes the same for tumor cells. The terms $h_{1}\;(cell^{-1})$ and $h_{2}\;(cell^{-1})$ act as constants for the fractional kill curves. For very small values of cells, *E* and *T*, the the kill rates are nearly linear but saturate if the populations of cells grow [29].

The model is made dimensionless by employing the following variables:

$$\bar{E}=\frac{E}{E_{0}},\bar{T}=\frac{T}{T_{0}},\bar{v}=\frac{v}{v_{0}},\bar{t}=nT_{0}t,\bar{s}=\frac{s}{nE_{0}T_{0}},\bar{p}=\frac{p}{nT_{0}},\bar{g}=\frac{g}{T_{0}},\bar{m}=\frac{m}{n},\bar{d}=\frac{d}{nT_{0}},$$ (3)

$$\bar{\alpha }=\frac{\alpha T_{0}^{-4/3}}{n},\bar{\beta }=\frac{\beta }{nT_{0}},{\bar{k}}_{E}=\frac{k_{E}v_{0}}{nT_{0}},{\bar{k}}_{T}=\frac{k_{T}v_{0}}{nT_{0}},{\bar{h}}_{1}=h_{1}E_{0},{\bar{h}}_{2}=h_{2}T_{0}.$$ (4)

Where *T*_0_, *E*_0_ and $v_{0}$ are reference values for *T*, *E* and *v* respectively.

The dimensionless model becomes:

$$\frac{d\bar{E}}{d\bar{t}}=\bar{s}+\frac{\bar{p}\bar{E}\bar{T}}{\bar{g}+\bar{T}}-\bar{m}\bar{E}\bar{T}-\bar{d}\bar{E}-\frac{{\bar{k}}_{E}\bar{E}\bar{v}}{1+{\bar{h}}_{1}\bar{E}}$$ (5)

$$\frac{d\bar{T}}{d\bar{t}}=\bar{\alpha }{\bar{T}}^{2/3}-\bar{\beta }\bar{T}-\bar{E}\bar{T}-\frac{{\bar{k}}_{T}\bar{T}\bar{v}}{1+{\bar{h}}_{2}\bar{T}}.$$ (6)

In the rest of this manuscript we drop the (*bar*) notation from all variables and parameters.

## 3 Model baseline parameters

**Fig 1:**
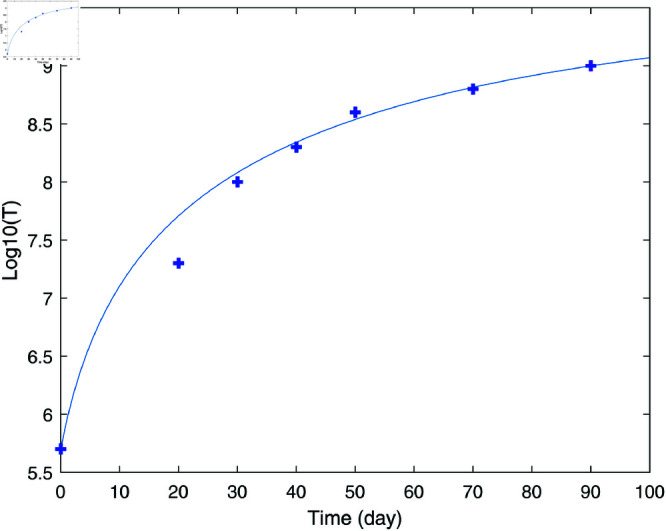
Fitting of tumor growth parameters (blue line) with data (cross point) for growth of BCL_1_ tumor in the spleens of chimeric mice [9].

The values of model parameters used for numerical simulations were carefully selected to reflect real situations and are shown in Table 1. The parameters α and β of tumor growth rate correspond to the normal growth of the tumor in the absence of an immune response. The experimental data correspond to the growth of BCL_1_ tumor in the spleens of chimeric mice as reported in [9]. Fig 1 shows the fitting of the data using the von Bertalanffy model. The fitting, carried out using the optimization code fmincon of MATLAB [30], yields values of $\alpha =51.55\;\frac{cell^{1/3}}{day}$ and $\beta =0.0363\;\frac{1}{day}$.

**Table 1:** Model baseline parameters [9, 11–13].

Parameter	Definition	Value	Unit	Dimensionless Value
*d*	Rate of natural demise of effector cells	0.0517	$\frac{1}{day}$	0.470
*g*	Steepness coefficient of the recruitment curve of effector immune cells	$2.02\times 10^{7}$	*cell*	20.2
*h*_1_	constant for chemotherapy drug effect on effector cell	$2\times 10^{-6}$	$\frac{1}{cell}$	2
*h*_2_	constant for chemotherapy drug effect on tumor cell	$2\times 10^{-6}$	$\frac{1}{cell}$	2
*k*_*E*_	Fractional effector cells kill by chemotherapy	$2\times 10^{-6}$	$\frac{1}{cell}$	2
*k*_*T*_	Fractional effector cells kill by chemotherapy	$2\times 10^{-6}$	$\frac{1}{cell}$	2
*m*	Degree of inactivation of effector cells by tumor cells	$3.3\times 10^{-11}$	$\frac{1}{cell.day}$	$3\times 10^{-4}$
*n*	Parameter of cancer cleanup	$1.1\times 10^{-7}$	$\frac{1}{cell}$	1
*p*	Degree of recruitment of maximum immune-effector cells in relation with cancer cells	0.1397	$\frac{1}{day}$	1.27
*s*	Growth rate of effector cells	$1.21\times 10^{4}$	$\frac{cell}{day}$	0.11
*v*	Effect of chemotherapy drugs on tumor	1.65	$\frac{cell}{day}$	15
*E*_0_	Base value for concentration of effector cells	10^6^	*cell*	—
*T*_0_	Base value for concentration of tumor cells	10^6^	*cell*	—
$v_{0}$	Base value for drug dose	10^3^	$\frac{mg}{m^{2}.day}$	—
α	Parameter of the growth model	51.55	$\frac{cell^{1/3}}{day}$	5.10
β	Parameter of the growth model	0.0363	$\frac{1}{day}$	0.33

The other model biological parameters (*s*,*p*,*g*,*d*,*m*,*n*) and the reference values $E_{0},T_{0}$ were also chosen to correspond to realistic ranges. We used most of the baseline parameter values reported in [9, 11–13].

As to the chemotherapy related parameters, values of chemotherapy drug dose *v* were varied in our numerical study. The literature indicates a wide range of variations from 0.0001 $\left(\frac{mg}{m^{2}.day}\right)$ to daily doses of thousands of $\left(\frac{mg}{m^{2}}\right)$ [31]. We have selected a base value of $v_{0}=10^{3}\;(\frac{mg}{m^{2}.day})$. The rest of the chemotherapy related parameters are difficult to estimate. Base values of *k*_*E*_ and *k*_*T*_ were taken to be 0.55 and 0.033 respectively as inspired from similar parameters reported in [16]. It should be noted that values of *k*_*E*_ are normally smaller than *k*_*T*_.

Moreover, the usefulness of the dimensionless model is to allow for a sensitivity analysis of the model parameters, especially for the chemotherapy-related ones. It can be seen that the parameter (*n*) representing the rate of tumor destruction by immune cells (Eq 2) was used to render the model parameters dimensionless. This parameter vary in the literature from 10^−7^ to $10^{-6}\;(\frac{1}{cell})$ [9, 11–14]. It can be concluded from (Eqs 3–4) that the dimensionless parameters $\left(s,p,m,d,k_{E},k_{T}\right)$ can be varied over a wide range which allow for a meaningful sensitivity analysis. As for the parameters *h*_1_ and *h*_2_, because of limited knowledge about their values their dimensionless values are varied over a wide range.

## 4 Equilibria existence and classification

In the following, we investigate the existence of non-trivial positive equilibria of the model (Eqs 5–6). Solving for the second Eq 6 yields

$$E=\frac{\alpha T^{2/3}-\beta T+\alpha h_{2}T^{5/3}-\beta h_{2}T^{2}-k_{T}Tv}{T(1+h_{2}T)}$$ (7)

Substituting Eq 7 in the steady state of Eq 5 yields the following polynomial of fourteen order:

$$F(U):=a_{0}+a_{1}U+a_{3}U^{3}+a_{4}U^{4}+a_{6}U^{6}+a_{7}U^{7}+a_{9}U^{9}+a_{10}U^{10}+a_{11}U^{11}\;\;+a_{12}U^{12}+a_{13}U^{13}+a_{14}U^{14}=0,$$ (8)

with *U* = *T*^1/3^. The coefficients of the polynomial are given in Appendix A. It can be seen that other than *a*_0_ and *a*_12_ which are positive, the rest of coefficients can either be positive or negative and no obvious relations can be deduced to define their signs. According to Descartes rule of sign, the maximum number of real and positive roots can be 14. However, any meaningful solution for *T*, must also satisfy the condition E≥0. The polynomial form of the steady-state questions (Eq 8) can be conveniently used to numerically solve for the steady-state solutions together with the use of Matcont package for bifurcation analysis [32].

In the following we start by analyzing the behavior of the model with no chemotherapy treatment.

## 5 Numerical simulations for the model with no chemotherapy treatment

In the absence of chemotherapy treatment (v=0), the model equilibria are still described by a polynomial of fourteen order (Eq 8) and the results in Appendix A still apply for the maximum number ogf positive real roots.

### 5.1 Phase space analysis

**Fig 2:**
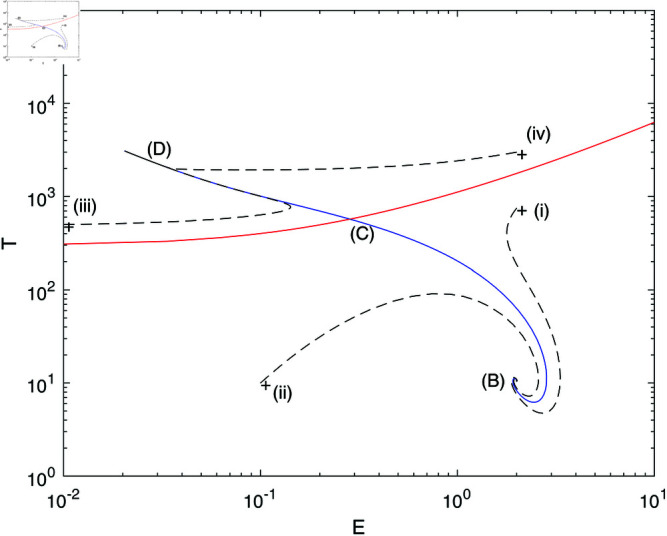
Phase portrait for the model with no chemotherapy v = 0 at the dimensionless parameter values in Table 1 and m=0.002. Stable manifold of saddle point (C) (blue); unstable manifold of saddle point C (red); (B), (C), and (D) are steady states; initial conditions (+) for transients (–) (denoted (i (iv)).

For the parameter values presented in Table 1, and *m* = 0.002, the polynomial (Eq 8) predicts three steady states in addition to the trivial solution $A(E=\frac{s}{d}=0.234,T=0)$. The non-trivial steady states are: B(E=1.994,T=10.56), C(E=0.285,T=571.64), and D(E=0.0205, T=3081.16). The steady state (*B*), classified as a stable spiral, is characterized by relatively low concentrations of tumor cells, and is referred to as the “dormant tumor” equilibrium point. In contrast, steady state (*D*), identified as a stable node, is marked by a high concentration of tumor cells and a low level of effector cells, which corresponds to a state of relatively “uncontrolled” tumor growth. Steady state (*C*), a saddle point has tumor cell levels situated between those of (*B*) and (*D*). The phase portrait is illustrated in Fig 2, where the one-dimensional stable manifold associated with the steady state (*C*) defines the basins of attraction for each of the respective attractors. Initial conditions (i) and (ii) in Fig 2 converge asymptotically towards the dormant tumor steady state (*B*), while initial conditions (iii) and (iv) allow the tumor to evade immune regulation, ultimately settling in the active steady state (*D*).

### 5.2 Hopf points

For the possibility of existence of periodic behavior (e.g. Hopf points) in the model, the Dulac-Bendixson criterion [33] can be used to investigate the existence of closed orbits for model Eqs 5–(6).

Consider the function ($M=\frac{1}{ET}$) and evaluate:

$$L:=\frac{\partial }{\partial E}(M\frac{dE}{dt})+\frac{\partial }{\partial T}(M\frac{dT}{dt}).$$ (9)

We have:

$$M\frac{dE}{dt}=\frac{s}{ET}+\frac{p}{g+T}-m-\frac{d}{T},$$ (10)

therefore

$$\frac{\partial }{\partial E}(M\frac{dE}{dt})=-\frac{s}{E^{2}T},$$ (11)

and

$$M\frac{dT}{dt}=\frac{\alpha }{ET^{1/3}}-\frac{\beta }{E}-1,$$ (12)

therefore

$$\frac{\partial }{\partial T}(M\frac{dT}{dt})=-\frac{\alpha }{3ET^{4/3}}.$$ (13)

This yields

$$L:=-(\frac{s}{E^{2}T}+\frac{\alpha }{3ET^{4/3}}).$$ (14)

One can observe that *L*<0 for positive values of *E*, *T*, and the model parameters. As a result, the Dulac-Bendixson criterion is met, which implies that Hopf bifurcations resulting in limit cycles are not possible.

We can go further and even prove a stronger result (as outlined in Appendix B): this competition model is unable to predict periodic behavior for all common tumor growth models referenced in the literature, including Mendelsohn, linear, surface, and Gompertz models. Furthermore, the logistic function has been previously analyzed in [9] and has been confirmed to lack the ability to demonstrate periodic behavior.

### 5.3 Bifurcation analysis

**Fig 3:**
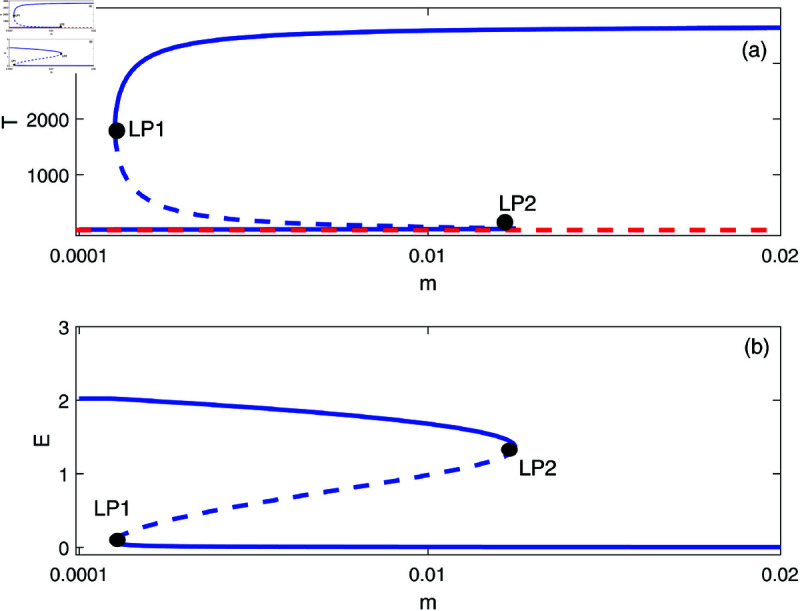
Bifurcation diagrams for the model with no chemotherapy at the dimensionless model parameters in Table 1. Solid line (stable branch); dashed line (unstable branch); dashed red line (unstable tumor-free equilibrium); LP (static limit point). (a) Variations of *T*; (b) Variations of *E*.

We proceed to conduct a bifurcation analysis, selecting (*m*) as the bifurcation parameter. This parameter represents the rate at which effector cells are rendered inactive due to their interactions with tumor cells. Preliminary studies [9, 20] as well as our own simulations have demonstrated the system’s sensitivity to variations in this parameter. Fig 3 illustrates the bifurcation diagram, which indicates the presence of hysteresis, with two limit points identified at *m* = 0.00112 and *m* = 0.0124. The following regimes can be noted: for 0<*m*<0.00112, solutions stabilize at the dormant state regime for any initial states. Within the range of 0.00112<*m*<0.0124, the system exhibits a coexistence of dormant and uncontrolled tumor states. For *m*>0.0124, the solutions of the system stabilize at the uncontrolled tumor regime for all initial conditions. In all the range of *m*, the tumor-free equilibrium (indicated by red dashed line) is unstable. At this point it is very much worthed to delineate the main differences of the model using the logistic growth rate studied in [9] and the same model with the Von Bertalanffy’s growth rate studied in this paper. The tumor-free equilibrium A(E=s/d,T=0) is always unstable for von-Bertalanffy’s associated model for any model parameters. The reason is the term *T*^2/3^ in the growth rate for which the derivative at *T* = 0 is not-defined, and some further analysis can show that the point is always unstable. This is not the case for logistic growth rate i.e. *aT*(1–*bT*). The tumor-free equilibrium can be unstable or stable depending on the sign of the term ((*a*–*b*)*d*–*s*) associated with the second eigenvalue of the Jacobian matrix at the tumor-free equilibrium. Details of mathematical derivations are given in Appendix C.

In order to illustrate this point, we take the same parameters for logistic model (*a* = 1.636 and $b=2.0\;\times \;10^{-3}$) that were used in the original work [9]. The rest of model parameters are taken from Table 1. In this case the term (a−b)d−s=.658 is positive and the tumor-free equilibrium is unstable. The bifurcation diagram will have exactly the same features as the one shown in Fig 3 and will not be plotted.

**Fig 4:**
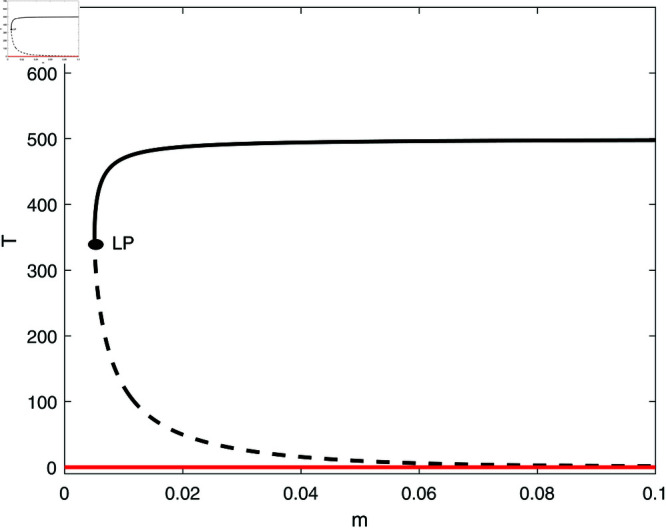
Bifurcation diagrams for the model with no chemotherapy and with logistic growth rate (aT(1–bT)) with $a\;=\;1.636,\;b\;=\;2\;\times \;10^{-3},\;s\;=\;0.35,\;d\;=\;0.15$ and the rest of dimensionless parameters in Table 1. solid line (stable branch); dashed line (unstable branch); solid red line (stable tumor-free equilibrium); LP (static limit point).

However, for different values of some parameters, for example for *s* = 0.35 and *d* = 0.15, the term (a−b)d−s=−0.1049 is negative and the tumor-free is stable. Fig 4 shows an example of such behavior. The bifurcation diagram has changed drastically. It can be seen that below the limit point, the system settles on the stable tumor-free equilibrium. Past the limit point there is coexistence of the tumor-free equilibrium with the uncontrolled tumor equilibrium. These fundamental differences will be elaborated on further in later section.

**Fig 5:**
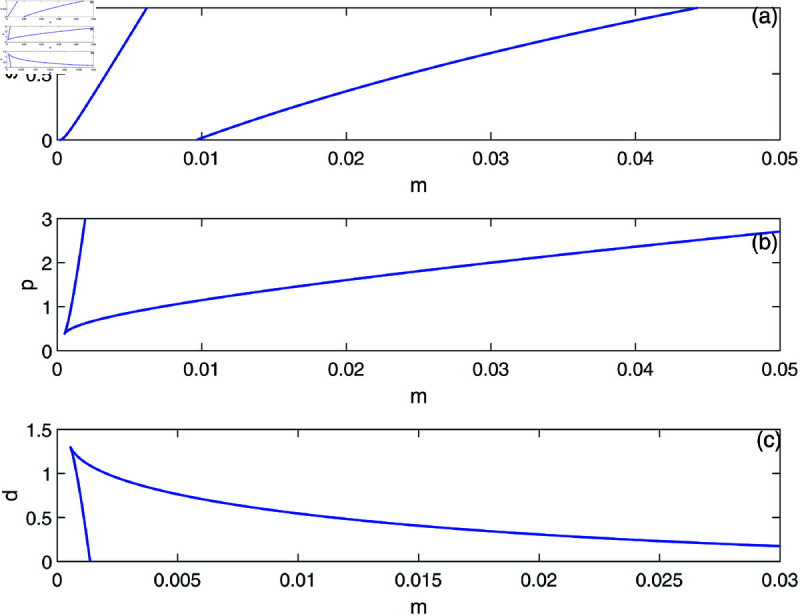
Two parameter continuation diagrams showing the loci of the limit point of Fig 3.

Going back to Fig 3 and in order to gain a deeper insight into its behavior, we delineate the various behavioral regions as function of key model parameters (Fig 5). Each branch depicted in Fig 5 represents a limit point, and a hysteresis phenomenon is anticipated between the two branches. Fig 5a illustrates the impact of the effector growth rate (*s*). The two branches converge at negative values of both (*s*) and (*m*). It is evident that bi-stability can occur for any growth rate (*s*). As (*s*) increases, the bi-stability increases in term of (*m*), highlighting the model’s sensitivity to tumor escape.

Fig 5b demonstrates the impact of the parameter *p*. We recall that *p* indicates the extent of effector cell recruitment in relation to cancer cells. The effect of *p* is comparable to the growth rate (*s*) of effector cells. However, hysteresis is only evident when *p* surpasses the critical point linked to the cusp.

The effect of the natural death rate *d* of effector cells is presented in Fig 5c. It is observed that hysteresis takes place only for (*d*) values that are lower than the cusp. With a reduction in (*d*), bi-stability can be observed across a wide array of (*m*) which again emphasizes the system’s vulnerability to tumor escape.

## 6 Numerical simulations for the model with chemotherapy treatment

In the following section we examine the dynamics of the model with the presence of chemotherapy (v≠0). We start by analyzing the possible existence of periodic behavior in the model.

### 6.1 Existence of Hopf points

The elements of the Jacobian matrix are obtained by taking the derivatives of Eqs 5–6:

$$J_{EE}=-d-mT+\frac{pT}{g+T}-\frac{k_{E}v}{(1+h_{1}E{)}^{2}}.$$ (15)

$$J_{ET}=-mE-\frac{pET}{(g+T{)}^{2}}+\frac{pE}{\left(g+T\right)}.$$ (16)

$$J_{TE}=-T$$ (17)

$$J_{TT}=-\beta -E+\frac{2\alpha }{3T^{1/3}}-\frac{k_{T}v}{(1+h_{2}T{)}^{2}}.$$ (18)

The existence of Hopf points [33] for the two dimensionless model (Eqs 5–6) is conditioned by:

$$J_{EE}+J_{TT}=0$$ (19)

$$J_{EE}J_{TT}>J_{ET}J_{TE}.$$ (20)

Using the steady state Eq 5 we can show that *J*_*EE*_ is reduced to

$$J_{EE}=-\frac{s}{E}+\frac{k_{E}vh_{1}E}{(1+h_{1}E{)}^{2}}.$$ (21)

Using the second steady state (Eq 6), and dividing by *T* and eliminating *T*^−1/3^, we can show that

$$J_{TT}=-\frac{\beta }{3}-\frac{E}{3}+\frac{k_{T}(2h_{2}T-1)v}{3(1+hT{)}^{2}}.$$ (22)

The first Hopf condition (Eq 19) is reduced to

$$-\frac{s}{E}-\frac{\beta }{3}-\frac{E}{3}+\frac{k_{E}h_{1}vE}{(1+h_{1}E{)}^{2}}+\frac{(2h_{2}T-1)v}{3(1+h_{2}T{)}^{2}}=0,$$ (23)

while the second condition (Eq 20) is equivalent to

$$ET(-m+\frac{gp}{(g+T{)}^{2}})>\frac{1}{3}(-\frac{s}{E}+\frac{h_{1}k_{E}vE}{(1+h_{1}E{)}^{2}})(\beta +E+\frac{k_{T}(1-2h_{2}T)v}{(1+h_{2}T{)}^{2}}).$$ (24)

One should note that the first three terms of Eq 23 are negative, implying that for Eq 23 to be satisfied, the condition

$$\frac{k_{E}h_{1}vE}{(1+h_{1}E{)}^{2}}+\frac{(2h_{2}T-1)v}{3(1+h_{2}T{)}^{2}}>0,$$ (25)

should be satisfied. This condition is evidently not satisfied when $h_{1}=h_{2}=0$. However, for non-zero values of *h*_1_ and /or *h*_2_, it is possible for the conditions specified in Eqs 23–24 to be met for some model parameter values, thus not excluding the existence of Hopf points.

### 6.2 Bifurcation analysis

**Fig 6:**
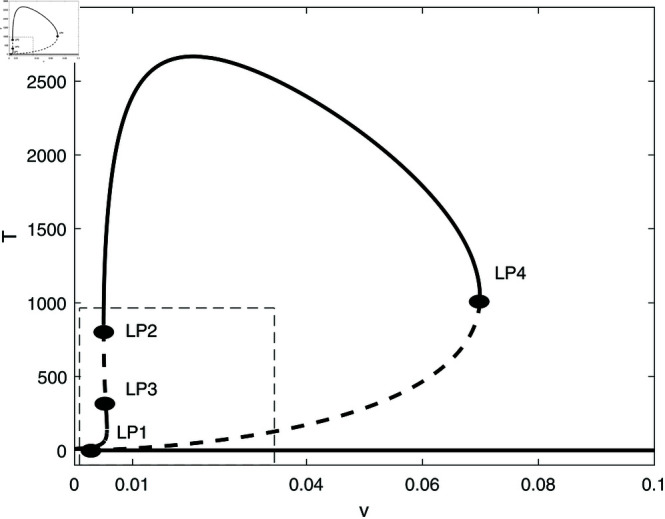
Bifurcation diagram for the model with chemotherapy at the dimensionless model parameters in Table 1. Solid line (stable branch); dashed line (unstable branch); LP (static limit point); The dashed rectangle is enlarged in Fig 7a and 7b.

**Fig 7:**
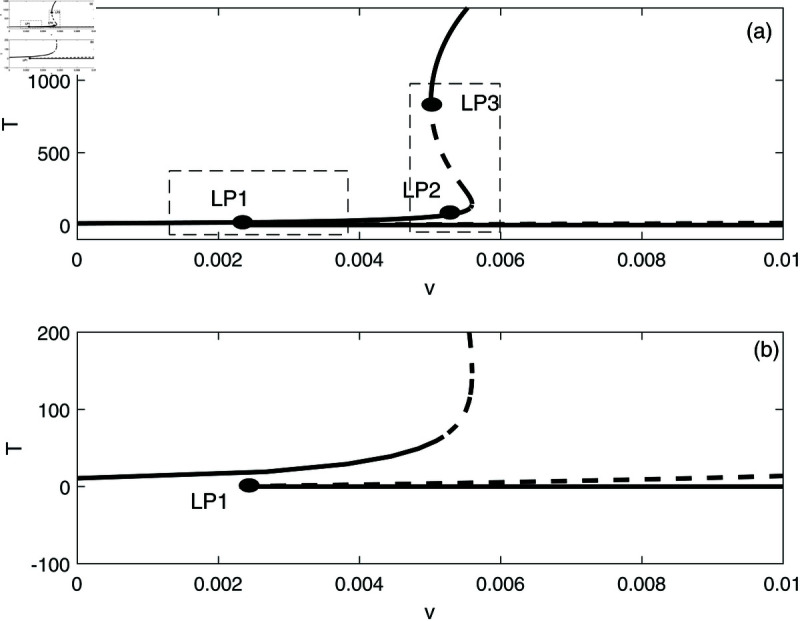
(a) Enlargement of the dashed rectangle in Fig 6; (b) Enlargement of the left dashed rectangle in Fig 7a. solid line (stable branch); dashed line (unstable branch); LP (static limit point).

**Fig 8:**
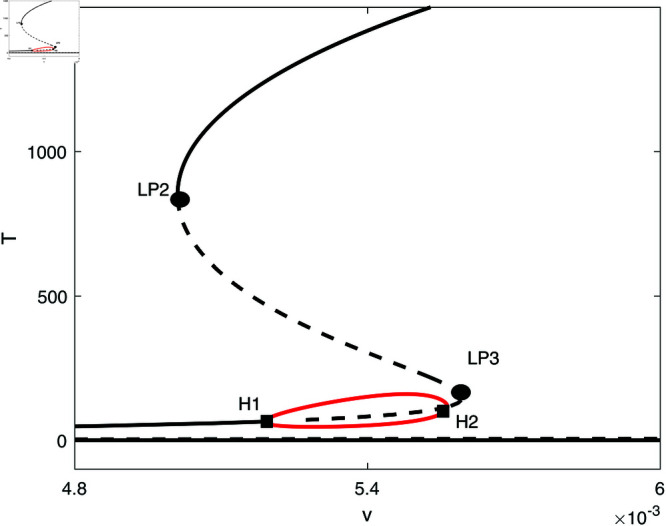
Enlargement of the dashed rectangle on the right of Fig 7a; solid line (stable branch); dashed line (unstable branch); red color (stable periodic branch); LP (static limit point); H (Hopf point).

**Fig 9:**
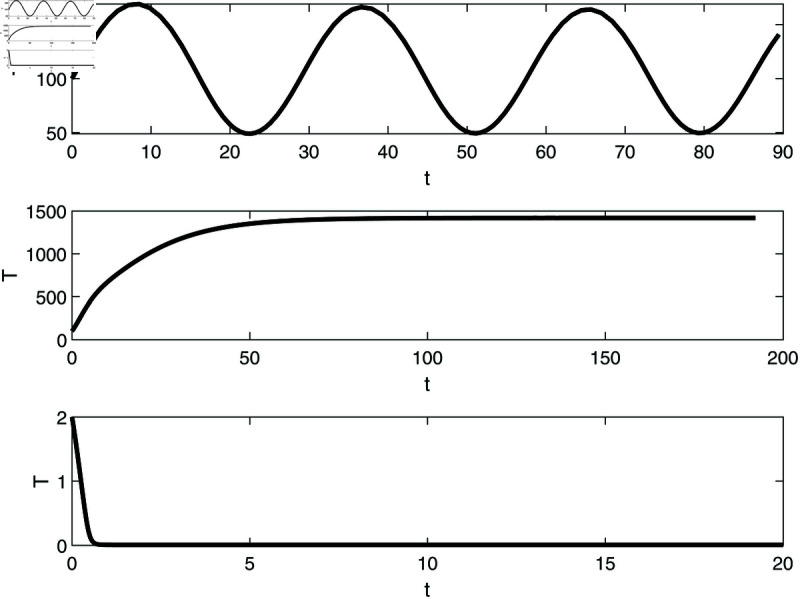
Simulations showing multi-stablity in Fig 8 at $v\;=\;5.40\times 10^{-3}$ and different initial conditions. (a) Start-up conditions (E,T)=(0.5,100) lead to sustained oscillations; (b) (E,T)=(0.49,100) lead to high tumor cells steady state. (c) (E,T)=(0.5,2) lead to the low steady-state.

In this section, we conduct bifurcation analyses of the model. It is convenient to select the chemotherapy dose (*v*) as the bifurcation parameter. The bifurcation diagram illustrated in Fig 6 corresponds to the parameter values listed in Table 1. Overall the diagram comprises an almost closed curve, with four static limit points *LP*_1_,*LP*_2_, *LP*_3_ and *LP*_4_. The maximum number of steady states can be shown to be five in addition to the tumor-free equilibrium. For example, for v=0.0054 we have the following steady states; (A)(E,T)=(0.05725,0.006717), (B)(E,T)=(0.06584,4.5217), (C)(E,T)=(0.6797,82.1690), (D)(E,T)=(0.3844,303.5360), and (E)(E,T)=(0.1143,1418.8600). (A) corresponds to very low tumor cells levels, (B) is a low tumor concentration, (C) and (D) can be considered as middle level tumor steady states while (E) is the very high tumor steady state. However, not all these equilibria are stable as can be seen in the following discussion. Going back to Fig 6 and in order to explain its behavior in more detail, the part indicated by a dashed rectangle in the left is enlarged in Fig 7a and 7b. It can be seen from Fig 7a and 7b that if the drug dose (*v*) is small and below the smallest limit point $\left(LP_{1};v=2.295\;\times \;10^{-3}\right)$, the tumor cells stabilize at the single steady state characterized by a relatively small tumor cells levels. When the drug dose increases past *LP*_1_, then Fig 7b shows the existence of three steady states, indicating the existence of bi-stability between the stable very low steady state and the stable middle steady state. The enlargement of the second dashed-rectangle (on the right) of Fig 7a, shown in Fig 8, reveals even more complex behavior. It can be sen that the previous regime of bi-stability extends from *LP*_1_ to *LP*_2_. However, there is the appearance of two Hopf points (*H*_1_ and *H*_2_). Stable periodic branches (in red color) connect the two Hopf points. Therefore for values of drug dose between *LP*_2_ and *H*_1_, three stable-steady states coexist: a very low tumor cells, a relatively low tumor cells, and the very high tumor cells levels. Different initial conditions can lead to either outcome. Between the two Hopf points, the system may settle on the very low steady state, on the very high tumor or stay on a state of oscillations. An example of such multi-stability is shown in Fig 9 for for $v=5.40\;\times \;10^{-3}$ and different initial conditions. Start-up conditions (E,T)=(0.5,100) lead to sustained oscillations, while a small decrease in immune cells from 0.5 to 0.49, lead to high tumor cells steady state. Small initial tumor levels (E,T)=(0.5,2) lead, on the other hand, to the low steady-state. In the tiny stable region between *H*_2_ and *LP*_3_ (Fig 8), there is again multi-stability between the very low tumor cells, the relatively low tumor cells, and the very high tumor cells steady state. In this region between *LP*_3_ and *LP*_4_ a bi-stability coexists between the very low steady state and the very high steady state. Finally, as the drug intensity increases, the high tumor concentration decreases and any drugs dose larger than *LP*_4_ leads to the tumor demise.

Next we examine the effect of the different model parameters on the various behavior observed so far in Figs 6, 7, 8. This can be done by showing the loci of the limit points and the Hopf points as function of the parameters’ values. The locations and crossing of the different limit and Hopf curves can give rise to additional bifurcation behavior. We focus on presenting only the results of key model parameters that have yielded bifurcation behavior other than the ones found in Figs 6, 7, 8.

**Fig 10:**
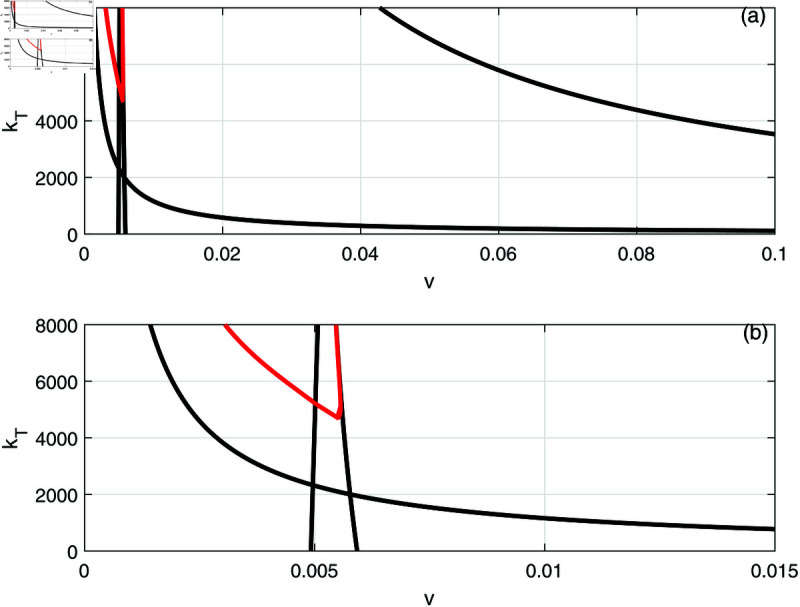
(a) Two parameter continuation diagrams $\left({v,k}_{T}\right)$ showing the loci of limit points (solid line) and Hopf point (red line) of Fig 6. (b) Enlargement of (a).

Fig 10a and 10b show the effect of the fractional tumor cell kill *k*_*T*_, with Fig 10b being an enlargement of Fig 10a. The solid lines indicate the loci of limit points while the red lines denote the loci of the Hopf points. It can be seen that the Hopf curve forms a minimum around *k*_*T*_ = 4200 and therefore for values of *k*_*T*_ smaller than the cusp, the model predicts four limit points but no Hopf points.

**Fig 11:**
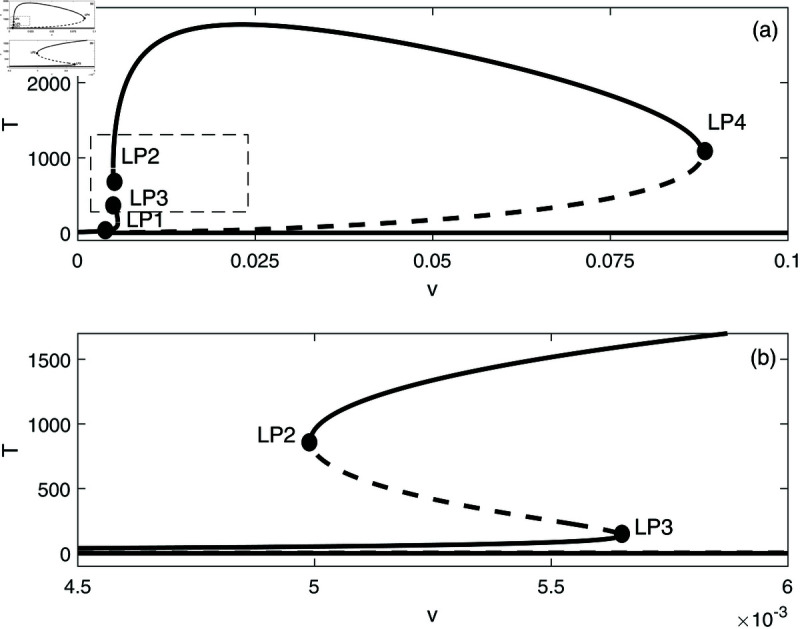
(a) Bifurcation diagram for the model with chemotherapy for $k_{T}$ = 4000 of Fig.10 and at the dimensionless model parameters in Table 1. LP (static limit point); solid line (stable branch); dashed line (unstable branch). (b) Enlargement of (a).

An example of this behavior is shown in Fig 11a and 11b for *k*_*T*_ = 3000. (Fig 11b being an enlargement of Fig 11a). The same patterns seen before in Figs 6, 7, 8 can be seen in Fig 11 except the absence of a periodic behavior.

**Fig 12:**
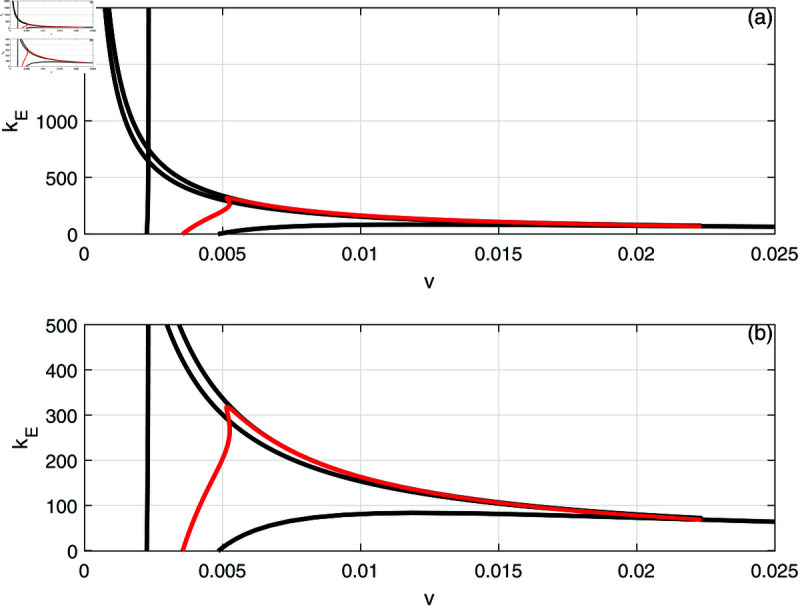
(a) Two parameter continuation diagrams $\left({v,k}_{E}\right)$ showing the loci of limit points (solid line) and Hopf point (red line) of Fig 6. (b) Enlargement of (a).

**Fig 13:**
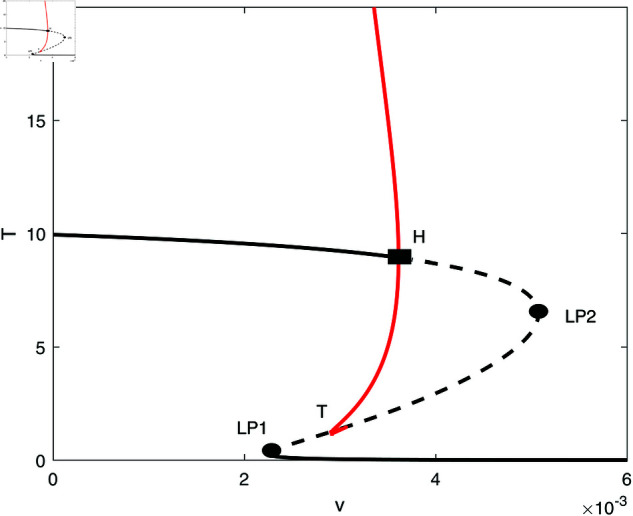
Bifurcation diagram for the model with chemotherapy for $k_{E}$ = 10 of Fig.12 and the dimensionless model parameters in Table 1. LP (static limit point); (T) point of collision of periodic branch with the unstable static branch; solid line (stable branch); dashed line (unstable branch); red curve (stable periodic branch).

Next we examine the effect of the fractional effector cell kill *k*_*E*_ shown in Fig 12. Here, the loci of Hopf points forms a maximum and therefore periodic behavior is expected below the maximum. Fig 13 shows an example of behavior for a small value of *k*_*E*_ = 10 that represents the case when the drug does not affect much the effector cells. The bifurcation diagram is characterized by the presence of two limit points and a HB point. Stable periodic branches emanate from the Hopf point and terminate as they collide at point (T) with the unstable static branch. It can be seen that drugs intensity below *LP*_1_ are unable to suppress the tumor. If *v* is increased past *LP*_1_ but below the point of termination (T), there is bi-stability between the very low tumor cells and the high active tumor cells. Between the termination point (T) and the Hopf point, there is multi-stability between three branches: the very low tumor concentration, the periodic branch and the high tumor branch. Values of drug dose larger than the Hopf point lead to the tumor demise.

**Fig 14:**
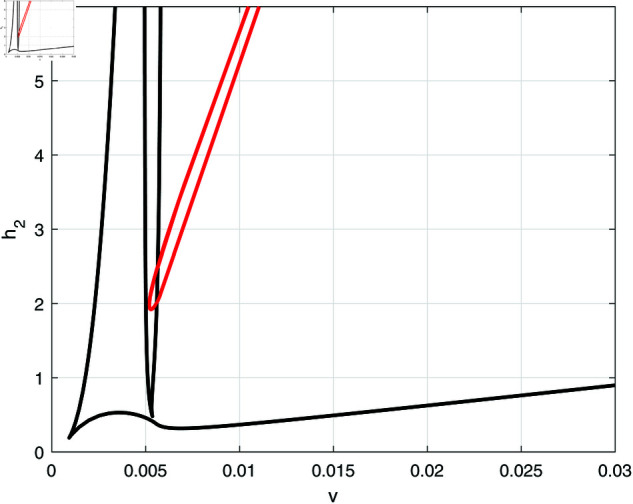
(a) Two parameter continuation diagrams $\left({v,h}_{2}\right)$ showing the loci of limit points (solid line) and Hopf point (red line) of Fig 6.

**Fig 15:**
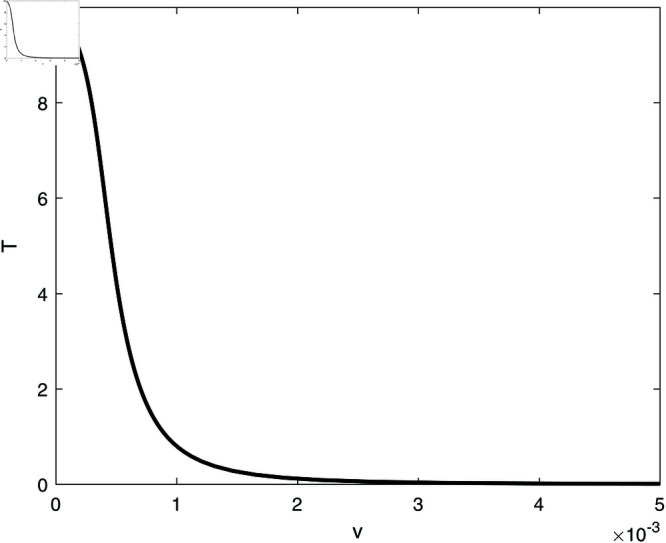
Bifurcation diagram for the model with chemotherapy for $h_{2}$ = 0 of Fig 14 and the dimensionless model parameters in Table 1. Solid line (stable branch).

The effect of the constant *h*_2_ (Eq 6) is shown in Fig 14. It can be seen that static limit points (and hence multiplicity) exist only for values of *h*_2_ larger than a certain value. We examine the case when *h*_2_ is zero which implies that the chemotherapy drug has a linear effect on the tumor. Fig 15 shows the bifurcation diagram for this case. No limit or Hopf point are excepted and the digram shows a simple monotonic decline of the tumor with the increase in the chemotherapy dose.

**Fig 16:**
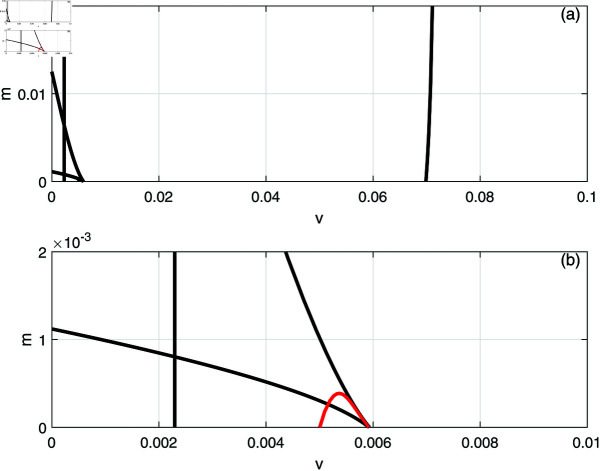
(a) Two parameter continuation diagrams (v,m) showing the loci of limit points (solid line) and Hopf point (red line) of Fig 6. (b) Enlargement of (a).

**Fig 17:**
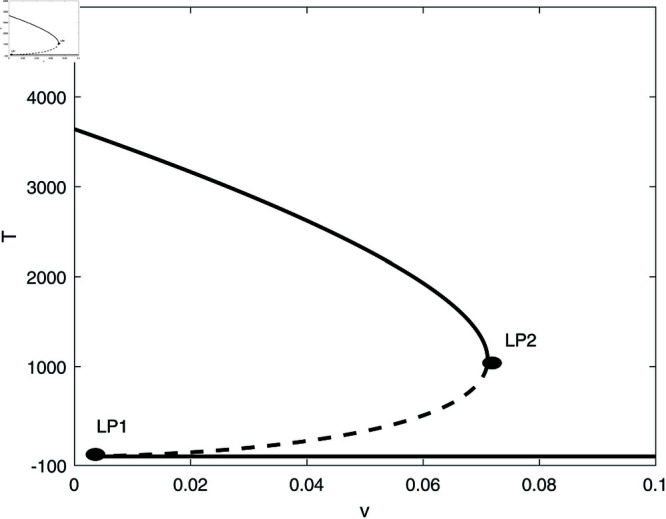
Bifurcation diagram for the model with chemotherapy for m = 0.02 of Fig 16 and the dimensionless model parameters in Table 1. LP (static limit point); solid line (stable branch); dashed line (unstable branch).

We turn now our attention to the effects of the biological parameters of the model. Two parameters yield additional and novel behavior. Fig 16 shows the effect of *m*, the rate of inactivation of effector cells by the tumor cells. Periodic behavior (red line) can be expected only below a critical value of *m*. Fig 17 shows a sample of behavior for *m* = 0.02. Only two limits points are expected. A classical hysteresis characterizes the behavior of the system. Below *LP*_1_ the drug is unable to eliminate the tumor. Between *LP*_1_ and *LP*_2_ the very low steady state coexists with the high tumor cells. Past *LP*_2_ the tumor is demised.

**Fig 18:**
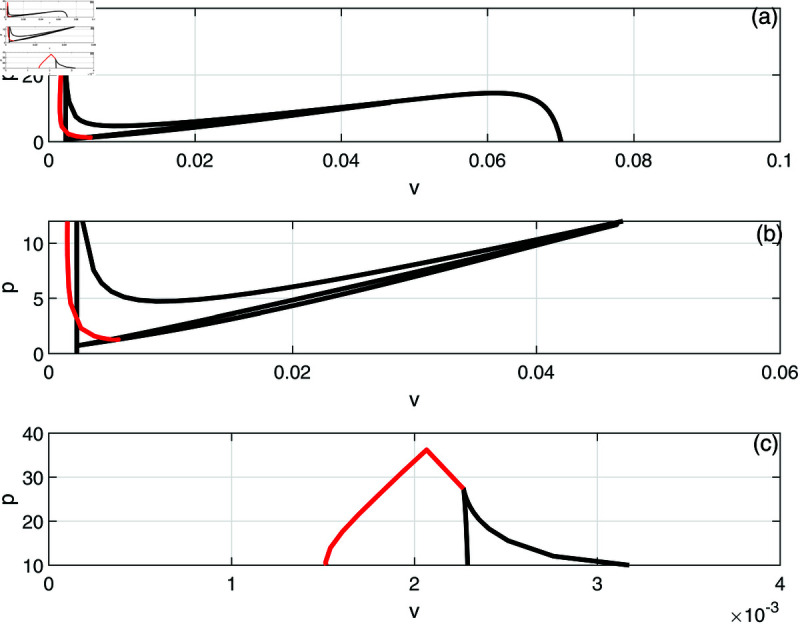
(a) Two parameter continuation diagrams (v,p) showing the loci of limit points (solid line) and Hopf point (red line) of Fig 6; (b) Enlargement of (a); (c) Enlargement of (a) for the tip of the diagram.

**Fig 19:**
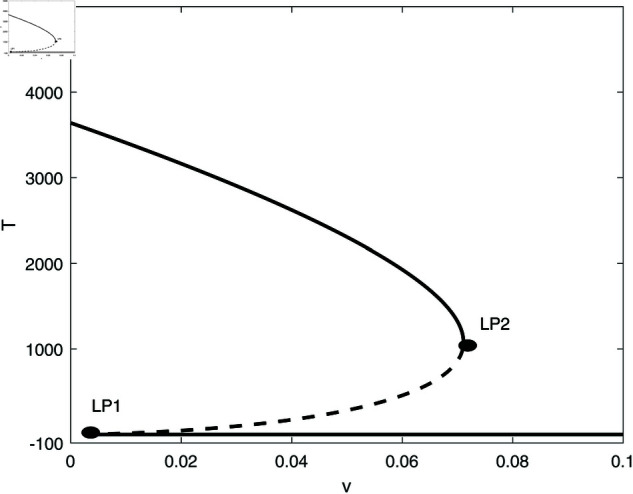
Bifurcation diagram for the model with chemotherapy for p = 20 of Fig 18 and the dimensionless model parameters in Table 1. LP (static limit point); H (Hopf point); solid line (stable branch); dashed line (unstable branch); red curve (sable periodic branch).

The last model parameter to be examined is the recruitment rate (*p*) of the effector cells. Fig 18 shows that Hopf points can exist only for values of *p* smaller than a critical value. The interesting feature of the figure is that for some range of *p* (Fig 18b) a Hopf point (red curve) exists before a limit point. Moreover, the enlargement in Fig 18c shows another interesting behavior which is the existence of Hopf points (red curve) without limit points. Fig 19 shows an example of the first behavior for *p* = 20. The diagram is characterized by the presence in this order of a Hopf point, limit point and a limit point. As the drug intensity increases, the tumor cells increase until the Hopf point. Since the branch between the two limit points is unstable, the behavior between Hopf point and the largest limit point (*LP*_1_) is characterized by the presence of an oscillatory behavior as the sole outcome of the effector-tumor cells interactions. Values of drug doses larger than *LP*_2_ causes the tumor to decrease until demise.

**Fig 20:**
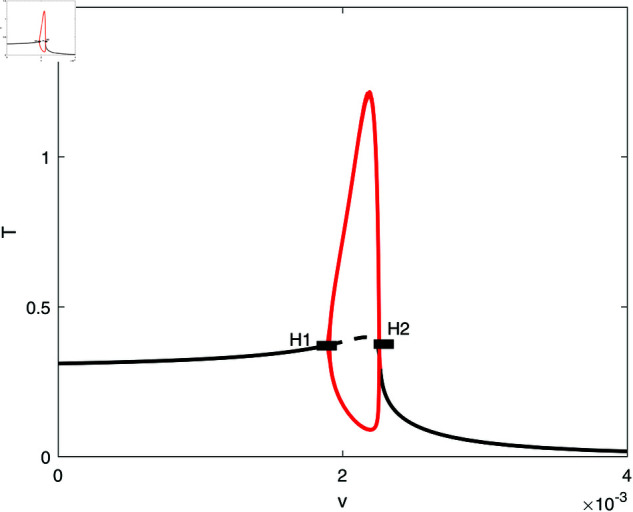
Bifurcation diagram for the model with chemotherapy for p = 30 of Fig 18 and the dimensionless model parameters in Table 1. LP (static limit point); H (Hopf point); solid line (stable branch); dashed line (unstable branch); red curve (sable periodic branch).

An example of the second behavior is shown for *p* = 30 in Fig 20. In this case two Hopf points are seen to occur and stable branches connect them. Similarly to the previous case of *p* = 20, oscillations are the only outcome between the two Hopf points. All the results of bifurcations found in the model are suitably summarized in Table 2.

**Table 2:** Summary of bifurcation behavior found in the diagrams.

Fig 3a and 3b	Key par.	Bif. par.	[0,*LP*_1_]	$\left[LP_{1},LP_{2}\right]$	$\geq LP_{2}$				
	(v = 0)	(m)	VLT	VLT,VHT	VLT				
Fig 4	Key par.	Bif. par.	[0,*LP*]	≥LP					
	(v,a,b)=(0,1.636,0.002)	(m)	TF	TF,VLT					
	(s,d)=(0.35,0.15)								
Figs 6, 7, 8	Key par.	Bif. par.	[0,*LP*_1_]	[*LP*_1_,*LP*2]	$\left[LP_{2},H_{1}\right]$	$\left[H_{1},H_{2}\right]$	$\left[H_{2},LP_{3}\right]$	$\left[LP_{3},LP_{4}\right]$	$\geq LP_{4}$
	-	(v)	VLT	VLT,MT	VT,MT,VHT	VLT,P,VHT	VLT,MT,VHT	VLT,VHT	VHT
Fig 11	Key par.	Bif. par.	[0,*LP*_1_]	[*LP*_1_,*LP*2]	$\left[LP_{2},LP_{3}\right]$	$\left[LP_{3},LP_{4}\right]$	$\geq LP_{4}$		
	(*k*_*T*_ = 4000)	(v)	VLT	VLT,MT	VT,MT,VHT	VLT,VHT	VHT		
Fig 13	Key par.	Bif. par.	$\leq LP_{1}$	[*LP*_1_,*T*]	[*T*,*H*]	≥H			
	(*k*_*E*_ = 10)	(v)	VHT	VLT,VHT	VLT,P,VHT	VLT			
Fig 17	Key par.	Bif. par.	$\leq LP_{1}$	$\left[LP_{1},LP_{2}\right]$	$\geq LP_{2}$				
	(m=0.02)	(v)	VHT	VLT,VHT	VLT				
Fig 19	Key par.	Bif. par.	≤H	[*H*,*LP*_1_]	$\geq LP_{1}$				
	(p=20)	(v)	VLT	P	VLT				
Fig 20	Key par.	Bif. par.	$\leq H_{1}$	$\left[H_{1},H_{2}\right]$	$\geq H_{2}$				
	(p=30)	(v)	VLT	P	VLT				

**Notes:** Bif. par.: Bifurcation parameter in the figure (x-axis). Key par.: All model parameter’s value are taken from Table (1), expect the value of the key parameter associated with the figure. MT: Medium tumor levels. P: periodic behavior. TF: Tumor-free equilibrium. VLT: Very low tumor levels . VHT: Very high tumor levels.  [–,–]: Range of the bifurcation parameter. X,Y: Indicates the coexistence of equilibria X and Y.

## 7 Discussion and biological implications

In the absence of chemotherapy treatment, the model with von Bertalanffy’s growth rate can predict some similar outcomes to the ones with the logistic growth model e.g. slight changes in model parameters can induce bi-stability in form of hysteresis. In actual circumstances, variations in parameter values are feasible as both effector and tumor cell populations are not uniform. Distinct subpopulations within these groups possess unique parameter values that influence their behavior. The model was shown to predict, similarly to the logistic growth, tumor dormancy and escape from immuno-regulation through a hysteresis bifurcation. However, there is a fundamental difference between the logistic growth and the von Bertalanffy’ model. In the former case, the tumor-free equilibrium can be stablized by change in some of the model parameters, through for instance, immunotherapy that can enhance the cytolytic potential of effector cells [11, 12] (parameter (m) in Eq 1). This is not the case for von Bertalnaffy’s model where the tumor-free disease is always unstable and the best outcome that could be predicted by the model is a very low tumor concentration steady state. Bistability was found to be accentuated by an increase in effector growth rate, a decrease in effect death rate, an increase in recruitment rate of effector cells and a decrease in the inactivation rate of effector cells.

For a bistable system, the location of the basin boundary is important in determining the outcome of the disease. Any treatment’s goal should be to move the system into the basin of attraction of the targeted equilibrium state. In the absence of chemotherapy, this goal can be attained by diminishing the tumor cell count through surgical techniques or radiation, and/or by augmenting the immune cell levels through adoptive cell transfer [11, 12, 21].

The model, in the absence of chemotherapy, was shown to be unable to predict an oscillatory behavior characterized by the continuous pursuit of competing cells. The findings reveal that the proposed model cannot predict periodic behavior for any set of parameter values. We even extended this result to other tumor growth rates expressions. It is “normally” advantageous for tumor cells to instigate fluctuations in the human body as this can lead to a temporary suppression of the immune system and an increased rate of tumor propagation [24]. This was clearly verified in some diseases such as malaria [34]. However, oscillatory behavior has not been confirmed in interactions between tumors and immune cells [24, 35] at least in the absence of chemotherapy. A plausible explanation is the possible risk of autoimmune reactions that may damage healthy cells. Therefore the lack of oscillations in the studied model, in the absence of chemotherapy, does not lower its quality despite its obvious simplicity.

The introduction of chemotherapy treatment, as external agent to the tumor-immune system, added considerable complexity to the types of behavior the model can predict. The complex interplay between the biological parameters of the model and the chemotherapy parameters can lead to a number of scenarios. Low doses of chemotherapy do not effectively suppress high tumor cell counts, while very high doses can result in tumor demise. When the drug intensity is situated between low and high levels, multi-stability is observed, characterized not only by hysteresis (coexistence of two stable steady states) but also multiple stable steady-states. In this latter instance, initial conditions or a temporarily weakened immune system can facilitate a transition from a very low tumor cell steady state to a medium steady state, circumventing a direct transition to the highest state. It should be noted that bi-stability/multi-stability may not be avoided in real situations since these phenomena were found to occur at small to moderate drug doses i.e. when the dimensionless drug dose was less than approximately 0.1, which corresponds to value of $100\;(\frac{mg}{m^{2}})$ per day, a value well within guidelines of most chemotherapy drugs [31]. Therefore even with chemotherapy placing the patient through the right basin boundary is critical to avoid re-growth of tumor. This could be achieved if immunotherapy is administered to a patient after chemotherapy which will cause the effector levels to increase allowing the system to move towards the intended equilibrium.

Moreover, the model predicted that the administration of chemotherapy can trigger oscillations. For some model parameters, the oscillations coexist with the static branches while in other situations and for some range of chemotherapy drug they are the only outcomes of the treatment. These oscillations are certainly unwanted and can cause harm to other body functions therefore they should be avoided, either by moving the drug dose outside the unwanted range or by placing the system in the right basin of attractions. The range of the oscillatory behavior was found to be reduced with the increase in the tumor cells lysis rate *m*, the recruitment rate *p* of effector cells, and fractional tumor kill cells *k*_*T*_. On the other hand, the range of oscillations decreases with increase in *k*_*E*_ and/or *h*_2_.

Furthermore, we established a significant finding: the inclusion of a saturation parameters (*h*_1_) and (*h*_2_) in the chemotherapy term $k_{E}vE/(1+h_{1}E)$ or in the tumor $\left(k_{T}vT/(1+h_{2}T\right)$ term is essential for the emergence of such oscillations. A linear effect $k_{E}vE$ or $k_{T}vT$ (i.e. $h_{1}=h_{2}=0$) does not produce any oscillatory behavior across any model parameters. Consequently, the oscillations are attributed not only to the presence of the chemotherapy agent (v≠0) but also to its type of impact on the reduction of tumor cells. Specifically, if the rates of effector and tumor cell reduction is directly proportional to the drug intensity, oscillations will not occur for any model parameter’s values.

A final note should be made about the limitations of this work. The model is certainly the simplest model that could be used to explain tumor-immune cells interactions. But out analysis has shown that this simple and classical model has quite a lot of blood in it. Furthermore, although the model parameters were taken from experimental settings, some of the found behavior associated with the chemotherapy needs to be validated against real life outcomes. Also in real life the effect of chemotherapy drug is not direct and includes some sort of delay. Including such delay in the expressions of the effects of chemotherapy will deserve further study.

## Supporting information

Appendix A Coefficients of polynomial of Eq 8 (PDF)

Appendix B Proof of Hopf points for arbitrary tumor growth rate. (PDF)

Appendix C Proof of stability of disease-free equilibrium for logistic growth. (PDF)

## References

[1] Bray F, Laversanne M, Sung H, Ferlay J, Siegel RL, Soerjomataram I, et al. Global cancer statistics 2022: GLOBOCAN estimates of incidence and mortality worldwide for 36 cancers in 185 countries. CA Cancer J Clin. 2024;74(3):229–63. doi: 10.3322/caac.21834 38572751

[2] Raghani NR, Chorawala MR, Mahadik M, Patel RB, Prajapati BG, Parekh PS. Revolutionizing cancer treatment: comprehensive insights into immunotherapeutic strategies. Med Oncol. 2024;41(2):51. doi: 10.1007/s12032-023-02280-7 38195781

[3] Liu B, Zhou H, Tan L, Siu KTH, Guan X-Y. Exploring treatment options in cancer: Tumor treatment strategies. Signal Transduct Target Ther. 2024;9(1):175. doi: 10.1038/s41392-024-01856-7 39013849 PMC11252281

[4] Murphy H, Jaafari H, Dobrovolny HM. Differences in predictions of ODE models of tumor growth: a cautionary example. BMC Cancer. 2016;16:163. doi: 10.1186/s12885-016-2164-x 26921070 PMC4768423

[5] Ghaffari Laleh N, Loeffler CML, Grajek J, Staňková K, Pearson AT, Muti HS, et al. Classical mathematical models for prediction of response to chemotherapy and immunotherapy. PLoS Comput Biol. 2022;18(2):e1009822. doi: 10.1371/journal.pcbi.1009822 35120124 PMC8903251

[6] Mathur D, Barnett E, Scher HI, Xavier JB. Optimizing the future: how mathematical models inform treatment schedules for cancer. Trends Cancer. 2022;8(6):506–16. doi: 10.1016/j.trecan.2022.02.005 35277375 PMC9117454

[7] Craig M, Jenner AL, Namgung B, Lee LP, Goldman A. Engineering in medicine to address the challenge of cancer drug resistance: from micro- and nanotechnologies to computational and mathematical modeling. Chem Rev. 2021;121(6):3352–89. doi: 10.1021/acs.chemrev.0c00356 33152247

[8] Brady R, Enderling H. Mathematical models of cancer: when to predict novel therapies, and when not to. Bull Math Biol. 2019;81(10):3722–31. doi: 10.1007/s11538-019-00640-x 31338741 PMC6764933

[9] Kuznetsov VA, Makalkin IA, Taylor MA, Perelson AS. Nonlinear dynamics of immunogenic tumors: parameter estimation and global bifurcation analysis. Bull Math Biol. 1994;56(2):295–321. doi: 10.1007/BF02460644 8186756

[10] Matzavinos A, Chaplain MAJ, Kuznetsov VA. Mathematical modelling of the spatio-temporal response of cytotoxic T-lymphocytes to a solid tumour. Math Med Biol. 2004;21(1):1–34. doi: 10.1093/imammb/21.1.1 15065736

[11] de Pillis LG, Radunskaya AE, Wiseman CL. A validated mathematical model of cell mediated immune response to tumor growth. Cancer Res. 2005;65(17):7950–8. 16140967 10.1158/0008-5472.CAN-05-0564

[12] de Pillis LG, Gu W, Radunskaya AE. Mixed immunotherapy and chemotherapy of tumors: modeling, applications and biological interpretations. J Theor Biol. 2006;238(4):841–62. doi: 10.1016/j.jtbi.2005.06.037 16153659

[13] Al-Tameemi M, Chaplain M, d’Onofrio A. Evasion of tumours from the control of the immune system: consequences of brief encounters. Biol Direct. 2012;7:31. doi: 10.1186/1745-6150-7-31 23009638 PMC3582466

[14] López AG, Seoane JM, Sanjuán MAF. A validated mathematical model of tumor growth including tumor-host interaction, cell-mediated immune response and chemotherapy. Bull Math Biol. 2014;76(11):2884–906. doi: 10.1007/s11538-014-0037-5 25348062

[15] Lestari D, Sari ER, Arifah H. Dynamics of a mathematical model of cancer cells with chemotherapy. J Phys Conf. Ser. 2019.

[16] Makhlouf AM, El-Shennawy L, Elkaranshawy HA. Mathematical modelling for the role of CD4+T cells in tumor-immune interactions. Comput Math Methods Med. 2020;2020:7187602. doi: 10.1155/2020/7187602 32148558 PMC7049850

[17] Rihan FA, Rajivganthi C. Dynamics of tumor-immune system with random noise. Mathematics. 2021;9(21):2707.

[18] Das A, Dehingia K, Ray N, Sarmah HK. Stability analysis of a targeted chemotherapy-cancer model. Math Model Control. 2023;3(2):116–26.

[19] Mirzaei N M, Tatarova Z, Hao W, Changizi N, Asadpoure A, Zervantonakis IK, et al. APDE model of breast tumor progression in MMTV-PyMT Mice. J Pers Med. 2022;9(12):938–50. 10.3390/jpm12050807 PMC9145520 35629230

[20] Bashkirtseva I, Chukhareva A, Ryashko L. Modeling and analysis of nonlinear tumor-immune interaction under chemotherapy and radiotherapy. Math Meth Appl Sci. 2022;12(5):807.

[21] Song G, Liang G, Tian T, Zhang X. Mathematical modeling and analysis of tumor chemotherapy. Symmetry. 2022;14(4):704.

[22] Feng X, Liu M, Jiang Y, Li D. Dynamics and stability of a fractional-order tumor-immune interaction model with B-D functional response and immunotherapy. Fractal Fract. 2023;7(2):200.

[23] Azizi T. Mathematical modeling of cancer progression. Appl Math. 2024;4(3):1065–79.

[24] Kareva I, Luddy KA, O’Farrelly C, Gatenby RA, Brown JS. Predator-prey in tumor-immune interactions: a wrong model or just an incomplete one? Front Immunol. 2021;12:668221. doi: 10.3389/fimmu.2021.668221 34531851 PMC8438324

[25] Von Bertalanffy L. Quantitative laws in metabolism and growth. Q Rev Biol. 1957;32(3):217–31. doi: 10.1086/401873 13485376

[26] Vaidya VG, Alexandro FJ Jr. Evaluation of some mathematical models for tumor growth. Int J Biomed Comput. 1982;13(1):19–36. doi: 10.1016/0020-7101(82)90048-4 7061168

[27] Heesterman BL, Bokhorst J-M, de Pont LMH, Verbist BM, Bayley J-P, van der Mey AGL, et al. Mathematical models for tumor growth and the reduction of overtreatment. J Neurol Surg B Skull Base. 2019;80(1):72–8. doi: 10.1055/s-0038-1667148 30733904 PMC6365230

[28] Kühleitner M, Brunner N, Nowak W-G, Renner-Martin K, Scheicher K. Best fitting tumor growth models of the von Bertalanffy-PütterType. BMC Cancer. 2019;19(1):683. doi: 10.1186/s12885-019-5911-y 31299926 PMC6624893

[29] Gardner SN. Amechanistic, predictive model of dose-response curves for cell cycle phase specific and nonspecific drugs. Cancer Res. 2000;60(5):1417–25. 10728708

[30] MATLAB. Version 9.4.0 (R2018a), Natick, MA, USA: The MathWorks Inc. 2018.

[31] McDonnell AM. Chemotherapeutic agents and their uses, dosages, and toxicities. https://www.cancernetwork.com/view/chemotherapeutic-agents-and-their-uses-dosages-and-toxicities. Accessed 2025 April 1.

[32] Dhooge A, Govaerts W, Kuznetsov YA. New features of the software MatCont for bifurcation analysis of dynamical systems. Math Comput Model Dyn Syst. 2008;14(2):147–75.

[33] Wiggins S. Introduction to applied nonlinear dynamical systems and chaos. New York: Springer. 1980.

[34] Smith LM, Motta FC, Chopra G, Moch JK, Nerem RR, Cummins B, et al. An intrinsic oscillator drives the blood stage cycle of the malaria parasite Plasmodium falciparum. Science. 2020;368(6492):754–9. doi: 10.1126/science.aba4357 32409472 PMC7518718

[35] Dorraki M, Fouladzadeh A, Salamon SJ, Allison A, Coventry BJ, Abbott D. On detection of periodicity in C-reactive protein (CRP) levels. Sci Rep. 2018;8(1):11979. doi: 10.1038/s41598-018-30469-8 30097610 PMC6086826

