# Supplementary material for: Bifurcation study of a tumor-immune system with chemotherapy
Source: PLoS One. 2025 Jul 3;20(7):e0327304. doi: 10.1371/journal.pone.0327304 (PMC12225868; doi:10.1371/journal.pone.0327304)
Supplement: Appendix A — (PDF) [file pone.0327304.s001.pdf]

A Appendix. Coefficients of polynomial of Eq.(8)

$$
\begin{aligned}
a_0 =& \alpha^2 dgh_1 \\
a_1 =& \alpha g(-h_1 s + k_E v + d(1 - 2h_1(\beta + k_T v))) \\
a_2 =& g(-s + (\beta + k_T v)(h_1 s - k_E v + d(-1 + \beta h_1 + h_1 k_T v))) \\
a_3 =& \alpha^2 h_1(d + 2dgh_2 + gm - p) \\
a_4 =& \alpha(-d(-1 + 2\beta h_1)(1 + 2gh_2) - p + k_E v - 2dh_1(1 + gh_2)k_T v + h_1(-s + 2p(\beta + k_T v)) + \\
& g(-2h_1 h_2 s + 2h_2 k_E v + m(1 - 2h_1(\beta + k_T v)))) \\
a_5 =& (-1 + \beta h_1)(\beta(d + 2dgh_2 + gm - p) + s + 2gh_2 s) + \\
& (-\beta(k_E + 2gh_2 k_E - 2h_1 k_T(d + dgh_2 + gm - p)) + \\
& k_T(-d(1 + gh_2) - gm + p + h_1 s + gh_1 h_2 s))v + \\
& k_T(-(1 + gh_2)k_E + h_1 k_T(d + gm - p))v^2 \\
a_6 =& \alpha^2 h_1(dh_2(2 + gh_2) + m + 2gh_2 m - 2h_2 p) \\
a_7 =& \alpha(-(-1 + 2\beta h_1)(1 + 2gh_2)m - 2h_1(1 + gh_2)k_T m v + \\
& dh_2(2 + gh_2 - 2h_1(\beta(2 + gh_2) + k_T v)) + \\
& h_2(-(2 + gh_2)(h_1 s - k_E v) + 2p(-1 + 2\beta h_1 + h_1 k_T v))) \\
a_8 =& (-1 + \beta h_1)(\beta(dh_2(2 + gh_2) + m + 2gh_2 m - 2h_2 p) + h_2(2 + gh_2)s) - \\
& (\beta(gh_2^2 k_E - 2h_1 k_T m + 2h_2(k_E - h_1 k_T(d + gm - p))) + \\
& k_T(m + h_2(d + gm - p - h_1 s)))v + k_T(-h_2 k_E + h_1 k_T m)v^2 \\
a_9 =& \alpha^2 h_1 h_2(2m + h_2(d + gm - p)) \\
a_{10} =& \alpha h_2(d(h_2 - 2\beta h_1 h_2) + h_2((-1 + 2\beta h_1)p - h_1 s + k_E v) + \\
& m(2 + gh_2 - 2h_1(\beta(2 + gh_2) + k_T v))), \\
a_{11} =& (-1 + \beta h_1)h_2(\beta(2m + h_2(d + gm - p)) + h_2 s) - h_2(\beta h_2 k_E + k_T m - 2\beta h_1 k_T m)v \\
a_{12} =& \alpha^2 h_1 h_2^2 m \\
a_{13} =& \alpha h_2^2 m - 2\alpha\beta h_1 h_2^2 m \\
a_{14} =& -\beta h_2^2 m + \beta^2 h_1 h_2^2 m
\end{aligned}
$$
