# Supplementary material for: Bifurcation study of a tumor-immune system with chemotherapy
Source: PLoS One. 2025 Jul 3;20(7):e0327304. doi: 10.1371/journal.pone.0327304 (PMC12225868; doi:10.1371/journal.pone.0327304)
Supplement: Appendix B — (PDF) [file pone.0327304.s002.pdf]

B Appendix. Proof of Hopf points for arbitrary tumor growth rate

Consider a model in dimensional form with arbitrary growth rate $r(T)$:

$$\frac{dE}{dt} = s + \frac{pET}{g+T} - mET - dE \quad (26)$$

$$\frac{dT}{dt} = r(T) - nET \quad (27)$$

Applying Dulac-Bendixson criterion [33] with the function ($M = \frac{1}{ET}$) and evaluating L,

$$L := \frac{\partial}{\partial E}(M\frac{dE}{dt}) + \frac{\partial}{\partial T}(M\frac{dT}{dt}). \quad (28)$$

Therefore we have:

$$L := -(\frac{s}{E^2T} - \frac{1}{E}\frac{d(\frac{r}{T})}{dT}). \quad (29)$$

If $\frac{d(\frac{r}{T})}{dT}$ is negative then L is negative and no Hopf points can occur.

Let evaluate the expression $\frac{d(\frac{r}{T})}{dT}$ for the most common expressions of $r(T)$ found in the literature [4].

- Mendelshon model: $r(T) = aT^b$ with $b < 1$ and $\frac{d(\frac{r}{T})}{dT} = a(b-1)T^{b-2}$ which is negative.
- Linear model: $r(T) = \frac{aT}{T+b}$ and $\frac{d(\frac{r}{T})}{dT} = \frac{-a}{(T+b)^2}$ which is also negative.
- Surface model: $r(T) = \frac{aT}{(T+b)^{1/3}}$ and $\frac{d(\frac{r}{T})}{dT} = -\frac{a}{3(T+b)^{4/3}}$ which is negative.
- Gompetz model: $r(T) = aTln(\frac{b}{T+c})$ and $\frac{d(\frac{r}{T})}{dT} = -\frac{a}{c+T}$ which is also negative.
