# Supplementary material for: Bifurcation study of a tumor-immune system with chemotherapy
Source: PLoS One. 2025 Jul 3;20(7):e0327304. doi: 10.1371/journal.pone.0327304 (PMC12225868; doi:10.1371/journal.pone.0327304)
Supplement: Appendix C — (PDF) [file pone.0327304.s003.pdf]

C Appendix. Proof of stability of disease-free equilibrium for logistic growth

Consider a model in dimensionless form with logistic growth rate:

$$\frac{dE}{dt} = s + \frac{pET}{g+T} - mET - dE \quad (30)$$

$$\frac{dT}{dt} = aT(1 - bT) - ET \quad (31)$$

The Jacobian matrix evaluated at the tumor-free equilibrium ($E = s/d, T = 0$) is

$$J = \begin{pmatrix} -d & -\frac{ms}{d} + \frac{ps}{dg} \\ 0 & a - b - \frac{s}{d}. \end{pmatrix}. \quad (32)$$

The eigenvalues are: $-d, \frac{(a-b)d-s}{d}$. Therefore, the tumor-free equilibrium is stable if $(a - b)d - s < 0$ and unstable otherwise.
